# Atopic dermatitis in Ethiopia: a systematic review and meta-analysis

**DOI:** 10.1093/inthealth/ihaf125

**Published:** 2025-11-03

**Authors:** Kidane Zereabruk, Woldu Aberhe, Assefa Iyasu, Abrha Hailay, Teklehaimanot Gereziher Haile, Gebreamlak Gebremedhn Gebremeskel, Kibrom Abera Alemayehu, Guesh Gebreayezgi, Teklay Guesh, Negasi Asres Mesfin, Guesh Mebrahtom

**Affiliations:** Department of Adult Health Nursing, School of Nursing, Aksum University, Aksum, Ethiopia; Department of Adult Health Nursing, School of Nursing, Aksum University, Aksum, Ethiopia; Department of Adult Health Nursing, School of Nursing, Aksum University, Aksum, Ethiopia; Department of Adult Health Nursing, School of Nursing, Aksum University, Aksum, Ethiopia; Department of Maternity and Neonatal Nursing, School of Nursing, Aksum University, Aksum, Ethiopia; Department of Adult Health Nursing, School of Nursing, Aksum University, Aksum, Ethiopia; Department of Health system, School of Public Health, Aksum University, Aksum, Ethiopia; Department of Epidemiology and Biostatistics, School of Public Health, Aksum University, Aksum, Ethiopia; Department of Epidemiology and Biostatistics, School of Public Health, Aksum University, Aksum, Ethiopia; Department of Epidemiology and Biostatistics, School of Public Health, Aksum University, Aksum, Ethiopia; Department of Adult Health Nursing, School of Nursing, Aksum University, Aksum, Ethiopia

**Keywords:** atopic dermatitis, Ethiopia, meta-analysis, prevalence, systematic review

## Abstract

Despite being a leading contributor to the global burden of skin disease there is no information on the national prevalence of atopic dermatitis in Ethiopia. Therefore, the purpose of this systematic review and meta-analysis was to estimate the pooled national prevalence of atopic dermatitis in Ethiopia. A computerized systematic search using multiple databases was performed in search of relevant English articles from the inception of the databases to 30 July 2023. It was done in accordance with the Preferred Reporting Items for Systematic review and Meta-Analysis (i.e. PRISMA) standard. R and R Studio software were used for statistical analysis. A random-effects model was used for calculating the pooled estimate of the prevalence of atopic dermatitis. Forest plots and tables were used to represent the data. We found 10 full-text observational studies with 25 920 participants suitable for the review after checking for inclusion and exclusion criteria. The overall pooled prevalence of atopic dermatitis in Ethiopia was 12.75% (95% CI 8.32 to 17.17%). The subgroup analysis found that the atopic dermatitis pooled prevalence was 7% (95% CI 3 to 10%), 17% (95% CI 4 to 30%) and 20% (95% CI 15 to 24%) in the Oromia region, Amhara region and Addis Ababa city administration, respectively. This study demonstrates that atopic dermatitis is highly prevalent in Ethiopia, affecting approximately one in eight individuals, with significant geographical variations. To effectively address this condition, it is crucial to implement awareness campaigns, prioritize specialized training for healthcare professionals, allocate resources to high-prevalence areas, establish a national registry for monitoring, develop community support programs and encourage further research, as there are currently few studies on the condition.

## Introduction

Atopic dermatitis (AD) is a chronic, non-contagious skin disease that significantly impacts the quality of life (QoL) of individuals globally. It is characterized by intense itching, scaly lesions, papules, fissures and crusting,^1^ and impacts the QoL of millions worldwide.^2^ This condition stands as a leading contributor to the global burden of skin diseases, with a global prevalence of 2.6%, affecting approximately 204.05 million people. This includes around 101.27 million adults (2.0%) and 102.78 million children (4.0%). Females are more frequently affected than males, with a prevalence of 2.8% (about 108.29 million) compared with 2.4% in males (approximately 95.76 million).^3^

Globally, skin diseases have garnered significant interest due to their commonality and potential for prevention and control, ranking as the 18th leading cause of disability-adjusted life years and the fourth leading cause of non-fatal disease burden in 2013.^4^

Although the prevalence of AD is increasing globally, particularly in urban areas of developing countries, comprehensive data on its national prevalence in Ethiopia are still severely lacking.^5^ Despite its importance, the prevalence of AD, particularly in developing countries like Ethiopia and among Ethiopians, remains poorly understood. Understanding the prevalence of AD in Ethiopia is crucial for several reasons: first, as urbanization accelerates in developing nations, the incidence of AD is rising, which may have significant public health implications; second, untreated or poorly managed AD can negatively impact individuals' QoL, leading to physical, emotional and social challenges. Additionally, the lack of awareness and insufficient healthcare resources further exacerbate the challenges faced by patients with AD in Ethiopia. The etiology of AD is multifactorial, with researchers suggesting that it may be triggered by environmental factors in genetically predisposed individuals, where heredity plays a crucial role in immune sensitization and allergy development.^6^ Additionally, early-life gastrointestinal factors may contribute to immune dysfunction linked to AD.^7,8^ AD is diagnosed based on clinical evaluation because no laboratory markers or definitive tests exist, with diagnostic criteria created to standardize patient enrollment in research studies.^2,9^ Our study aims to address critical questions concerning the national prevalence of AD in Ethiopia. Specifically, we aim to estimate the pooled prevalence of AD across the regions of the nation and evaluate the variability in prevalence rates. This attempt is essential in providing the much-needed data that can inform public health initiatives effectively. Current research underscores the importance of having robust epidemiological data to guide healthcare policies and strategies. In light of this, our study aims to fill a notable research gap regarding the prevalence of AD in Ethiopia through a systematic review and meta-analysis. By employing rigorous methodologies, we aspire to contribute valuable insights to the existing literature, which can ultimately enhance clinical practices, inform healthcare policies and direct future research efforts aimed at improving the QoL for individuals affected by AD in the country.

## Methods

### Study protocol

The systematic review and meta-analysis adheres to the Preferred Reporting Items for Systematic Reviews and Meta-Analyses (PRISMA) guidelines^10^ (Supplementary Material 1).

### Systematic review registration

This systematic review and meta-analysis has been registered in the International Prospective Register of Systematic Reviews (PROSPERO) database (CRD42020186932).

### Study setting

This systematic review and meta-analysis was conducted in Ethiopia, a country in eastern Africa.

### Study design

We conducted a systematic review and meta-analysis that encompassed all studies reporting the prevalence of AD in Ethiopia. These studies were exclusively observational and cross-sectional in nature.

### Search strategy and information sources

Using databases such as PubMed/MEDLINE, Embase, Science direct, Scopus, Google Scholar, Web of Science, Cochran Library, Africa Wide Information, the WHO Afro Library and Africa Index Medicus, we conducted a thorough review of articles that discuss the prevalence of AD in Ethiopia from inception of the database to 30 July 2023. The existence of prior systematic reviews or protocols on the topic of interest was investigated. The following keywords were searched using BOOLEAN (AND/OR) operators to combine search terms: ‘Atopic dermatitis’, ’prevalence’, ‘incidence’, ‘Ethiopia’, ‘systematic review and meta-analysis’, and combinations of these terms were used. The existence of prior systematic reviews or protocols on the topic of interest was investigated by searching various databases. The databases searched include the Cochrane Database of Systematic Reviews, Joanna Briggs Institute Database of Systematic Reviews and Implementation Reports, the National Health Center Review and Dissemination Database, Health Technology Assessment, the Campbell Collaboration Library and Evidence for Policy and Practice Information Center. The search of the aforementioned databases confirmed that there was no systematic review and/or protocol on the topic of interest. Additionally, we searched the reference lists of eligible articles to identify additional relevant studies.

### Data extraction and quality assessment

Three authors (AI, WA and AH) extracted the data independently using a prepiloted template developed in a Microsoft Excel spreadsheet. To identify possibly qualifying papers, two reviewers (KZ and GM) independently checked out the titles, abstracts of every reference obtained and full-text search results. The data extraction included the following: title, author name(s), study design, year of data collection, year of publication, sample size, number of cases to AD, response rate, study area or setting, study region, study quality score and prevalence rate. Additionally, appropriate sampling techniques, consistent data collection techniques, recorded quality control methods and a representative sample size were all considered as indicators of study quality.

### Criteria for considering studies for the review

Inclusion criteria

Design: all published observational studies.

Population: study participants are from all age groups.

Publication status: only peer-reviewed articles.

Settings: hospital-based studies.

Language: this review solely took into account English-language articles.

Method of diagnosis: no restriction on methods of diagnosis.

Intervention(s)/exposure(s): not applicable.

Outcome: prevalence of AD.

Exclusion criteria

The following studies were excluded from consideration: case reports and case series studies, as well as studies lacking the pertinent information required to calculate the prevalence of AD in Ethiopia.

### Quality assessment and risk of bias in individual studies

The methodological quality of the included studies was evaluated using the Newcastle–Ottawa Scale (Supplementary Material 2). The Newcastle–Ottawa Scale was designed to assess the quality of non-randomized studies in meta-analyses. This scale is primarily formulated by a star allocation system, assigning a maximum of 10 stars for the risk of bias in three areas: a selection of study groups (4 or 5 stars), comparability of groups (2 stars) and ascertainment of the outcome of interest or exposure (3 stars). No validation study provides a cut-off score for rating low-quality studies; a priori, we arbitrarily established that 0–3, 4–6 and 7–10 stars would be considered as at high, moderate and low risk of bias, respectively.^11^ Differences between the two reviewers were settled via dialogue and discussion.

For each included study, we estimated the precision (C) or margin of error, considering the sample size (SS) and the observed prevalence (Pr) of AD from the formula:


$${\rm SS} \frac{{z}^{2}*p* ( 1 - p )}{d^{2,}}$$

where *Z* is the *z* value fixed at 1.96 across studies (corresponding to 95% CI). The desirable margin of error is ≤5% (0.05).

### Data management

Based on the inclusion and exclusion criteria, a tool was developed to guide the screening and selection process. The tool was piloted and revised before data extraction began. The search results were uploaded to EndNote 20 software first to remove duplicates.

### Data analysis and presentation of results

A meta-analysis was performed to estimate the pooled prevalence of patients with AD using R version 4.3.1 and R studio version 1.524 software. Forest plots were drawn to visualize the combined prevalence of AD and the extent of statistical heterogeneity between studies. The pooled estimate was computed using the ‘meta prop’ command. Heterogeneity across the studies was assessed by Cochrane’s Q test and I^2^ statistic. The I^2^ statistic ranges from 0 to 100%. The I^2^ statistic, with values of 25%, 50% and 75%, is representative of low, medium and high heterogeneity, respectively.^12^ There was high heterogeneity between the included studies. Therefore, we used a random-effects model to estimate the overall pooled national prevalence of AD. A subgroup analysis was summarized by geographic regions where the study was conducted. Tables and forest plots with 95% CIs are used to present the results. A funnel plot was used to assess publication bias. The results of this review are reported based on the PRISMA guidelines.^10^

### Study selection and data collection process

Studies that were performed in Ethiopia and that reported on the prevalence of AD were chosen for the meta-analysis. Three independent reviewers (KAA, WA and AH) screened the titles and abstracts of all initially identified studies based on predefined selection criteria. In any case of disagreement, a decision was reached through consensus or consultation with an independent author (KZ). In instances of missing information, the corresponding author of the study under inspection was contacted to solicit the necessary details; an E-mail was dispatched for supplementary information before determining the exclusion of that study. For studies that occurred in multiple publications, we used the one that was the most recent, comprehensive and had the largest sample size. For surveys that appeared in a single article with several surveys conducted at various times, we treated each survey as a separate study.

### Data items

Data on general information—author(s), data collection year, publication year, region, study characteristics (study design, setting, case, sample size, prevalence rate, response rate) and quality assessment—were extracted.

### Outcomes and prioritization

The primary outcome is the prevalence of AD in Ethiopia.

### Data synthesis

To estimate the estimated national prevalence of AD in Ethiopia, a meta-analysis was conducted. The findings are illustrated through forest plots. Subgroup analysis focused on the different regions within Ethiopia where the studies were carried out. Given the heterogeneity among the studies, a random-effects model^13^ was applied to ascertain the overall national prevalence of AD in Ethiopia. Heterogeneity was explored using Cochrane’s Q and quantified by I^2^ statistics.^12^ The outcomes are conveyed as proportions along with their respective 95% CIs. The results of this review are reported based on the PRISMA guidelines.

## Results

### Screening flow

**Figure 1.**
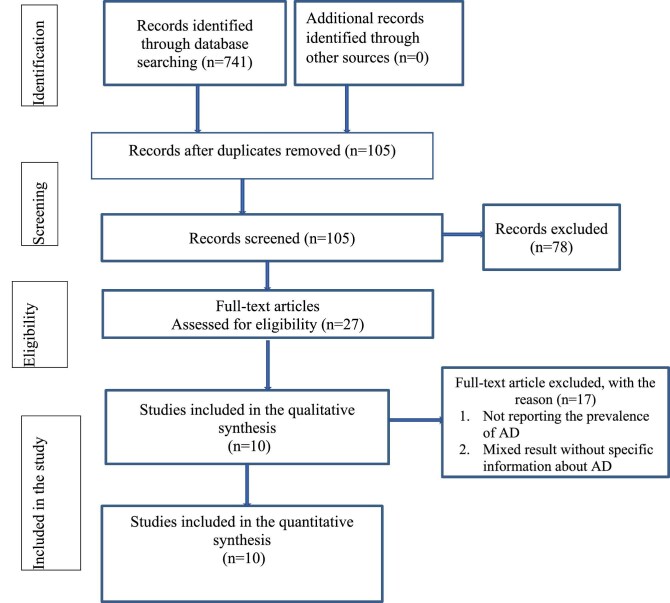
Flowchart diagram showing the selection of articles for the systematic review and meta-analysis of atopic dermatitis (AD) in Ethiopia, 2024.

A schematic representation of the process that was used to identify and select the studies that were included is illustrated in Figure 1. The included databases and number of included studies thereof were PubMed (243), Science Direct (53), Google Scholar (241), Cochrane Library (11), Africa Wide Information (74), the WHO Afro Library (14), African Journal Online (80), Web of Science (14), Scopus (4) and African Index Medicus (7). Based on the predefined criteria and quality assessment, 636 duplicates were identified and removed. Subsequently, we screened 105 titles and abstracts and excluded 78 irrelevant papers. Of the 27 articles assessed for eligibility, 17 did not report a prevalence value for AD. In the end, this systematic review and meta-analysis included 10 full-text articles and 25 920 participants of all ages. The detailed steps of the screening process are shown in Figure 1.

### Study characteristics

In this meta-analysis, 10 studies were included. Three (30%) of the studies were from the Oromia region, two (20%) of the studies were from the Amhara region and two (20%) of the studies were from Addis Ababa city administration. The rest of the studies were from the SNNR, Tigray and Somalia regions. All of the studies had a cross-sectional study design. The sample sizes ranged from 105 to 12 876 patients. The highest and lowest prevalence of AD was reported from Amhara and Oromia with 23.6% and 1.2%, respectively. The quality score of each primary study, based on the Newcastle–Ottawa Scale quality assessment criteria, showed no considerable risk; therefore, all the studies were considered in this systematic review and meta-analysis (Table 1).

**Table 1.** Characteristics of studies included in the systematic review and meta-analysis of atopic dermatitis in Ethiopia, 2024

Authors	Data collection year	Publication year	Region	Area/setting	SD	Cases	Sample size	Prevalence rate	Response rate	Quality assessment
Kelbore et al.^14^	2017	2019	SNNPR	Hospital	Cs	212	1704	12.40%	100%	9
Haileamlak et al.^15^	2003	2004	Oromia	Hospital	Cs	350	7915	4.40%	100%	9
Mehanna et al.^16^	2017	2018	Oromia	Hospital	Cs	91	541	16.80%	100%	9
Kelbore et al.^17^	2014	2015	Tigray	Hospital	Cs	45	470	9.60%	98.50%	9
Tegegne^18^	2016	2018	Amhara	Hospital	Cs	218	923	23.60%	100%	9
Abdela et al.^19^	2018	2020	Amhara	Hospital	Cs	68	661	10.28%	100%	9
Hassan et al.^20^	2020	2022	Somalia	Hospital	Cs	46	340	13.50%	96.70%	8
Yemaneberhan et al.^21^	1996	2004	Oromia	Hospital	Cs	153	12 876	1.20%	95%	8
Aschalew et al.^22^	2020	2022	Addis Ababa	Hospital	Cs	17	105	16.20%	100%	7
Gashaw et al.^23^	2020	2022	Addis Ababa	Hospital	Cs	82	385	21.30%	100%	9

Cs: cross-section; SD: study design; SNNPR: Southern Nations Nationalities and Peoples' Region.

### The pooled prevalence of AD in Ethiopia

**Figure 2.**
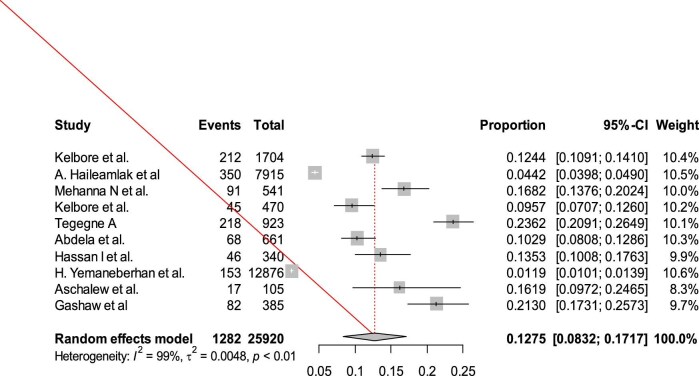
Forest plot of the pooled prevalence of atopic dermatitis in Ethiopia, 2024.

**Figure 3.**
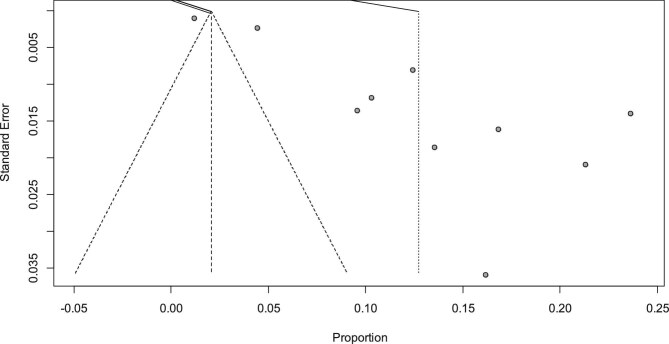
Funnel plot indicating the presence of publication bias.

In this review, the overall pooled prevalence of AD in Ethiopia was 12.75% (95% CI 8.32 to 17.17%). The heterogeneity test was checked and it was I^2^=99%, indicating that there is high heterogeneity (Figure 2). A leave-one-out sensitivity analysis was conducted to determine if the results of a particular study significantly influenced the overall estimated national prevalence of AD in Ethiopia. Nevertheless, all the outcomes of this sensitivity analysis fell within the 95% CI boundaries of the overall estimated national prevalence (8.32–17.17%), suggesting that no individual study had a significant impact on the observed national prevalence of AD (Supplementary Material 3). To assess potential publication bias, a funnel plot was utilized, revealing asymmetry and implying the existence of unpublished articles that might alter the overall estimated national prevalence of AD (Figure 3).

### Subgroup analysis of AD by study region of the country

According to the subgroup analysis, the prevalence of AD was 7% in the Oromia region (95% CI 3 to 10%), 17% in the Amhara region (95% CI 4 to 30%) and 20% in Addis Ababa city administration (95% CI 15 to 24%).

## Discussion

This systematic review and meta-analysis was performed to show the pooled estimates of nationwide results for AD in Ethiopia. AD is a prevalent chronic skin condition that has a detrimental effect on patients' QoL in terms of health-related aspects such as mental, emotional and physical functioning.^14^ In Ethiopia, there has never been a nationwide systematic review or prevalence study of AD. Hence, the final analysis of this systematic review and meta-analysis indicates a national pooled prevalence of AD in Ethiopia of 12.75% (95% CI 8.32 to 17.17%). The prevalence of AD varied among the studies in our study, ranging from 1.20%^15^ in the Oromia region to 23.6%^16^ in the Amhara region of Ethiopia. One possible explanation for this could be a complex link between genetic, environmental and health-seeking behavior-related factors.^17,18^ This result is lower compared with a systematic review and meta-analysis conducted in Asia (0.96–22.6%) and Europe (1.2–17.1%).^19^ Additionally, it is lower than the prevalence reported in a single study survey conducted in Denmark (21.3%),^20^ as well as being lower than that recorded for a study conducted in Oregon (17.2%)^21^ and that from a nationwide survey study conducted in Korea (26.5%).^22^ This could be explained by genetic and environmental-related factors, variation in healthcare access and awareness, variation in diagnostic criteria and methodology used in different studies, as well as different demographic characteristics of the population such as socioeconomic status, urbanization and age distribution that may contribute to the variation in the prevalence rates.^23–25^

However, the pooled prevalence of AD for Ethiopia of 12.75% calculated in the current study is higher than the prevalence of AD reported in an Italian systematic review and meta-analysis (10.2%),^26^ the US national survey of AD, which reported a prevalence of 7.3% (95% CI 5.9 to 8.8%)^27^ and a study conducted in Japan among Japanese adults, which reported a prevalence of 3.0%.^28^

This could be explained by various factors such as genetic predisposition, environmental factors like climate difference, exposure to allergens, access to healthcare services, differences in diagnostic criteria, lifestyle, differences among the populations and differences in the methodologies and sampling techniques used in those studies.^25,29,30^

To examine the heterogeneity of the studies included in this review, we conducted a subgroup analysis based on the regions of the country. We found varying pooled prevalences of AD in Oromia, Amhara and Addis Ababa city administration. This discrepancy could be due to various factors such as genetic predisposition, access to healthcare, lifestyle differences and variations in allergen exposure, hygiene practices and socioeconomic status among the populations living in these regions.^31–33^ Therefore, the disparity in prevalence rates emphasizes how crucial it is to consider population characteristics and regional considerations when evaluating epidemiological data on AD.

Although AD is one of the most common dermatological disorders and significantly impacts individuals' QoL, there is a limitation of studies worldwide, particularly among the adult population in sub-Saharan countries like Ethiopia. The epidemiology of AD varies considerably based on age, gender, prevalence measures, diagnostic criteria, genetic predisposition, access to healthcare, lifestyle differences, allergen exposure, hygiene practices and socioeconomic status among populations, and its distribution is uneven across geographical areas.^3,32,34^

The findings of this systematic review and meta-analysis may contribute to our understanding of the prevalence of AD in Ethiopia, and this may provide an input for the global and regional estimation of AD. In addition, it can significantly affect clinical practices by improving care for patients with AD, enhancing preventive measures, as well as serving as crucial health and safety benchmarks. These consolidated prevalence data on AD can help in developing effective prevention strategies and advocating for resources that support individuals in overcoming obstacles to lower the burden of this condition.

The observed heterogeneity in prevalence across different regions of Ethiopia underscores the need for targeted interventions and further research to identify the underlying factors contributing to these variations. This is the first systematic review and meta-analysis of the national pooled prevalence of AD in Ethiopia. However, limitations of the included studies, such as differences in study design, diagnostic criteria and sample sizes, should be acknowledged. Standardization of research methodologies and diagnostic criteria for future studies will enhance the comparability of findings and contribute to a more accurate assessment of AD prevalence in Ethiopia. To the best of our knowledge, this is the first systematic review and meta-analysis of the overall estimated national prevalence of AD in Ethiopia. The results provide key insights into the epidemiology of AD in the country, offering guidance for public health strategies and future research initiatives. However, this review has certain limitations: conducting subgroup analysis across all regions of the nation proved challenging due to statistical limitations and a paucity of studies. Additionally, this review did not include the factors contributing to the condition. Another limitation of our review was the presence of publication bias.

## Conclusions

This study reveals that AD is highly prevalent in Ethiopia, affecting approximately one in eight individuals and exhibiting significant geographical variations. To effectively address this public health issue, it is essential to implement targeted awareness campaigns, standardize diagnostic criteria and methodologies across healthcare facilities and prioritize specialized training for healthcare professionals. Additionally, resources should be allocated to high-prevalence areas, and a national registry for monitoring AD should be established. Conducting regional health assessments to identify contributing factors and developing community support programs are also crucial. By encouraging further research, we can enhance understanding of the condition. By taking these comprehensive actions, we can significantly reduce the burden of AD and improve the QoL for affected individuals in Ethiopia.

## Supplementary Material

ihaf125_Supplemental_Files

## Data Availability

The data analyzed during the current meta-analysis are available from the corresponding author upon reasonable request.
