# Supplementary material for: Atopic dermatitis in Ethiopia: a systematic review and meta-analysis
Source: Int Health. 2025 Nov 3;18(3):361–8. doi: 10.1093/inthealth/ihaf125 (PMC13154830; doi:10.1093/inthealth/ihaf125)
Supplement: ihaf125_Supplemental_Files [file ihaf125_supplemental_files.zip › supplementary Material 3.docx]

**A leave-one-out sensitivity analysis among included studies showing if the overall estimated prevalence of Atopic Dermatitis was greatly affected by the result of a single study**

proportion 95%-CI

Omitting Kelbore et al. 0.1280 [0.0785; 0.1776]

Omitting A. Haileamlak et al 0.1372 [0.0920; 0.1823]

Omitting Mehanna N et al. 0.1231 [0.0747; 0.1714]

Omitting Kelbore et al. 0.1312 [0.0823; 0.1801]

Omitting Tegegne A 0.1146 [0.0732; 0.1560]

Omitting Abdela et al. 0.1305 [0.0813; 0.1796]

Omitting Hassan, I et al. 0.1268 [0.0776; 0.1761]

Omitting H.Yemaneberhan et al. 0.1406 [0.1003; 0.1810]

Omitting Aschalew et al. 0.1244 [0.0764; 0.1724]

Omitting Gashaw et al 0.1182 [0.0732; 0.1631]

**Pooled estimate 0.1275 [0.0832; 0.1717]**

**Appendix**: Forest plot for the sensitivity analysis of the included studies in the systematic review and meta-analysis of atopic dermatitis in Ethiopia.
